# Safety and feasibility of direct return to the ward after transcatheter aortic valve replacement: a prospective observational study

**DOI:** 10.3389/fcvm.2026.1773793

**Published:** 2026-03-11

**Authors:** Zhi Li, Qianer Li, Yanlin Ye, Lulu Liu, Yuanyuan Wang, Beiyao Lu, Miao Chen

**Affiliations:** 1 Department of Cardiovascular Surgery, West China Hospital/West China School of Nursing, Sichuan University, Chengdu, Sichuan, China; 2 Outpatient Department, West China Hospital, Sichuan University, Chengdu, Sichuan, China; 3 Department of Cardiovascular Surgery, West China Hospital, Sichuan University, Chengdu, Sichuan, China

**Keywords:** early discharge, intensive care unit, patient safety, postoperative care, propensity score matching, transcatheter aortic valve replacement, ward admission

## Abstract

**Background:**

With increasing procedural volumes and improved safety, transcatheter aortic valve replacement (TAVR) programs are exploring strategies to streamline postoperative care. This study aimed to evaluate the safety and feasibility of direct return to the cardiovascular surgery ward after TAVR.

**Methods:**

This prospective observational study enrolled patients who underwent TAVR between January and April 2024 and were directly admitted to the cardiovascular surgery ward postoperatively if they met predefined criteria. A historical cohort of patients treated between January and December 2023 who met the same eligibility criteria but were admitted to the CICU served as the control group. Propensity score matching was used to ensure comparability between groups. The primary outcome was the composite early safety endpoint defined by Valve Academic Research Consortium-3 (VARC-3). Secondary outcomes included 30-day major complications (per VARC-3), mortality, readmission, postoperative delirium, unplanned ICU transfer, and postoperative length of stay.

**Results:**

Of the 168 patients who underwent TAVR between January and April 2024, 130 (77.4%) were directly transferred to the ward. In the historical cohort, 231 patients were included as controls. After propensity score matching, 95 patients were included in each group. The 30-day composite early safety event rate was 8.4% in the ward group and 11.6% in the CICU group (OR = 0.7, 95%CI: 0.3–1.7), with no significant difference. Secondary outcomes, including major complications, mortality, readmissions, postoperative delirium, and unplanned ICU transfers, were comparable between groups. Median postoperative length of stay was significantly shorter in the ward group (4 vs. 6 days; mean difference 1.9 days, 95%CI: 1.3–2.5).

**Conclusions:**

Direct ward transfer after TAVR appears safe and feasible in selected patients and is associated with a shorter hospital stays compared to routine CICU admission.

## Introduction

Transcatheter aortic valve replacement (TAVR) has become one of the main treatments for patients with aortic valve disease (1, 2). Although TAVR is minimally invasive, it is predominantly performed in older patients with multiple comorbidities, which makes case selection more challenging and increases procedural risk (3, 4). As a result, these patients are vulnerable to serious complications and require careful postoperative monitoring (5–7). Traditionally, patients are admitted to the cardiac intensive care unit (CICU) or coronary care unit (CCU) for 24 to 48 h after TAVR to ensure hemodynamic stability and manage procedure-related complications.

In recent years, advancements in TAVR technology and increased operator experience have significantly reduced complication rates, laying a solid foundation for optimizing postoperative care pathways. Meanwhile, the global expansion of TAVR—now increasingly applied to intermediate- and low-risk populations (8–11)—has led to a marked rise in procedural volumes (12), potentially placing mounting pressure on ICU capacity across many centers. At our center, for instance, annual TAVR volume increased more than 15-fold since its introduction in 2014, from 24 to 364 cases in 2023, with daily volumes occasionally exceeding 10. However, our CICU has only 20 beds, which also accommodate over 3,000 cardiac surgeries annually. In this context, direct ward transfer after TAVR—without routine ICU admission—may help improve procedural efficiency and optimize resource utilization.

Minimalist TAVR protocols—characterized by transfemoral access, conscious sedation or local anesthesia, and early mobilization—have generated increasing interest in simplifying postoperative care (13–16). Several studies have shown that bypassing ICU admission is safe for appropriately selected low-risk patients (17–19). However, current evidence is largely limited to transfemoral TAVR performed under minimalist conditions. Data remain limited regarding the safety and feasibility of direct ward admission in more complex settings—such as procedures involving alternative access routes or general anesthesia. Moreover, standardized ward-based monitoring strategies to support safe recovery in these scenarios have not been well established.

Therefore, this study aimed to evaluate the safety and feasibility of direct admission to the cardiovascular surgery ward following TAVR. We hypothesized that direct ward transfer would not increase the risk of postoperative complications, unplanned ICU transfer, 30-day readmission, or mortality, and might reduce the incidence of postoperative delirium and length of hospital stay compared to conventional CICU admission.

## Material and methods

### Design and setting

This prospective observational study was conducted in the department of cardiovascular surgery at a tertiary teaching hospital between January and April 2024. All consecutive patients undergoing TAVR were included during this period, and those meeting eligibility criteria were directly admitted to the general ward postoperatively. The study was approved by the institutional biomedical research ethics committee (IRB No. 20241474), and informed consent was obtained from all participants. For comparison, a historical cohort of patients who underwent TAVR and were admitted to the CICU between January and December 2023 was retrospectively analyzed. As this analysis was based on routinely collected clinical data, the requirement for informed consent was waived by the IRB.

Until the end of 2023, all patients undergoing TAVR at this center were routinely admitted to the CICU postoperatively. To serve as a control group, a historical cohort of patients treated between January and December 2023 and admitted to the CICU after TAVR was retrospectively analyzed. As this retrospective analysis involved only routinely collected clinical data, the requirement for informed consent was waived by the IRB.

### Participants

The participants were patients who underwent elective TAVR with transfemoral or transapical access by the same surgical team. Beginning in January 2024, patients were eligible for direct admission to the general ward postoperatively if they met all of the following criteria: (1) no requirement for invasive respiratory support prior to the procedure; (2) hemodynamic stability during the procedure; (3) absence of major vascular complications, new-onset arrhythmias, or any other intraoperative complications; and (4) successful extubation in the operating room or post-anesthesia care unit (PACU).

Patients meeting the same criteria who were admitted to the CICU between January and December 2023 were included as historical controls. To minimize potential selection bias, only those with a CICU stay of ≤48 h were included, ensuring comparability with ward-group patients who were expected to have stable postoperative recovery.

Accordingly, participants were categorized into two groups: the ward group (directly admitted to the cardiovascular surgery ward after TAVR) and the CICU group (admitted to the CICU after TAVR).

### Study procedures

All procedures were performed by the same surgical team using standardized operative and perioperative protocols throughout the study period. In our center, all patients underwent preoperative evaluation by a multidisciplinary Heart Team that typically includes cardiac surgeons (primary operators with established experience in structural heart interventions), dedicated imaging specialists (echocardiography and CT assessment), and anesthesiologists, with additional specialists involved as needed depending on clinical complexity and comorbidities.

For patients in the ward group, routine monitoring was conducted in the PACU following TAVR. After 30 min to 60 min of observation, patients were transferred directly to the cardiovascular surgery ward. This ward is a standard general ward without invasive monitoring or telemetry, and the nurse-to-patient ratio was 1:8 during daytime hours and 1:22 at night. To ensure patient safety during the early postoperative period following TAVR, a three-component enhanced ward monitoring protocol was implemented in addition to routine care during the first 24 hours after surgery. This protocol consisted of: (1) Enhanced observations, with vital sign assessments and clinical evaluation every 15 minutes for the first 2 hours, every 30 minutes for the subsequent 4 hours, and hourly thereafter. Monitoring included assessments of consciousness level, heart rate and rhythm, blood pressure, oxygen saturation, pain levels, and inspection of catheter access sites for early detection of complications. In addition, a standard 12-lead ECG was performed once daily after the procedure for rhythm assessment. (2) Enhanced examinations, consisting of two additional arterial blood gas analyses: one immediately after returning to the ward and another 4-6 h postoperatively to detect early physiological derangements. (3) Enhanced manpower, involving assignment of an additional nurse was assigned during the evening shift (18:00–22:00) on scheduled TAVR procedure days (typically Wednesdays and Saturdays) to support the increased monitoring needs and ensure the effective implementation of the protocol.

Patients in the CICU group received standard postoperative intensive care, including mechanical ventilation support (if needed), continuous electrocardiographic monitoring, invasive arterial and central venous pressure monitoring, frequent laboratory testing and blood gas analyses, and continuous bedside nurse presence for real-time surveillance. A standard 12-lead ECG was also recorded daily after the procedure as part of routine rhythm assessment.

### Study outcomes

The primary outcome was the composite early safety endpoint defined according to the Valve Academic Research Consortium-3 (VARC-3) criteria (20). This included freedom from all-cause mortality, any stroke, VARC type 2–4 bleeding, major vascular complications, access-related or cardiac structural complications, stage 3 or 4 acute kidney injury, moderate or severe aortic regurgitation, new permanent pacemaker implantation, and any surgery or intervention related to the device within 30 days.

Secondary outcomes included 30-day major complications (per VARC-3), 30-day hospital readmission, postoperative delirium, unplanned ICU transfer, and postoperative length of stay (LOS).

### Statistics

Propensity score matching (PSM) was performed to minimize selection bias and confounding between the ward and CICU groups. Propensity scores were estimated using logistic regression based on the following covariates: age, sex, body mass index (BMI), EuroSCORE II, New York Heart Association (NYHA) functional class, comorbidities, prior cardiac surgery or intervention, type of aortic valve disease, procedural access route, and procedure duration. A 1:1 nearest-neighbor matching without replacement was conducted, using a caliper width of 0.2 standard deviations of the logit of the propensity score. Covariate balance between matched groups was assessed using absolute standardized mean differences (SMD), with an SMD <10% considered indicative of acceptable balance.

Continuous variables were expressed as the mean with standard deviations (SD) or median with interquartile range (IQR). Categorical variables are summarized as frequencies and percentages. Comparisons between groups were performed both in the original cohort and propensity-matched cohort. Continuous variables were analysed using the independent samples *t*-test or the Mann–Whitney *U* test, while categorical variables were compared using Pearson's chi-squared test, chi-squared test with Yates's continuity correction, or Fisher's exact test. Additional subgroup analyses stratified by valve disease type were performed. A two-sided *P* value less than 0.05 was considered statistically significant. Data analyses were conducted using SPSS version 24.0 (IBM Inc., Armonk, NY, USA).

## Results

### Patient characteristics

**Figure 1:**
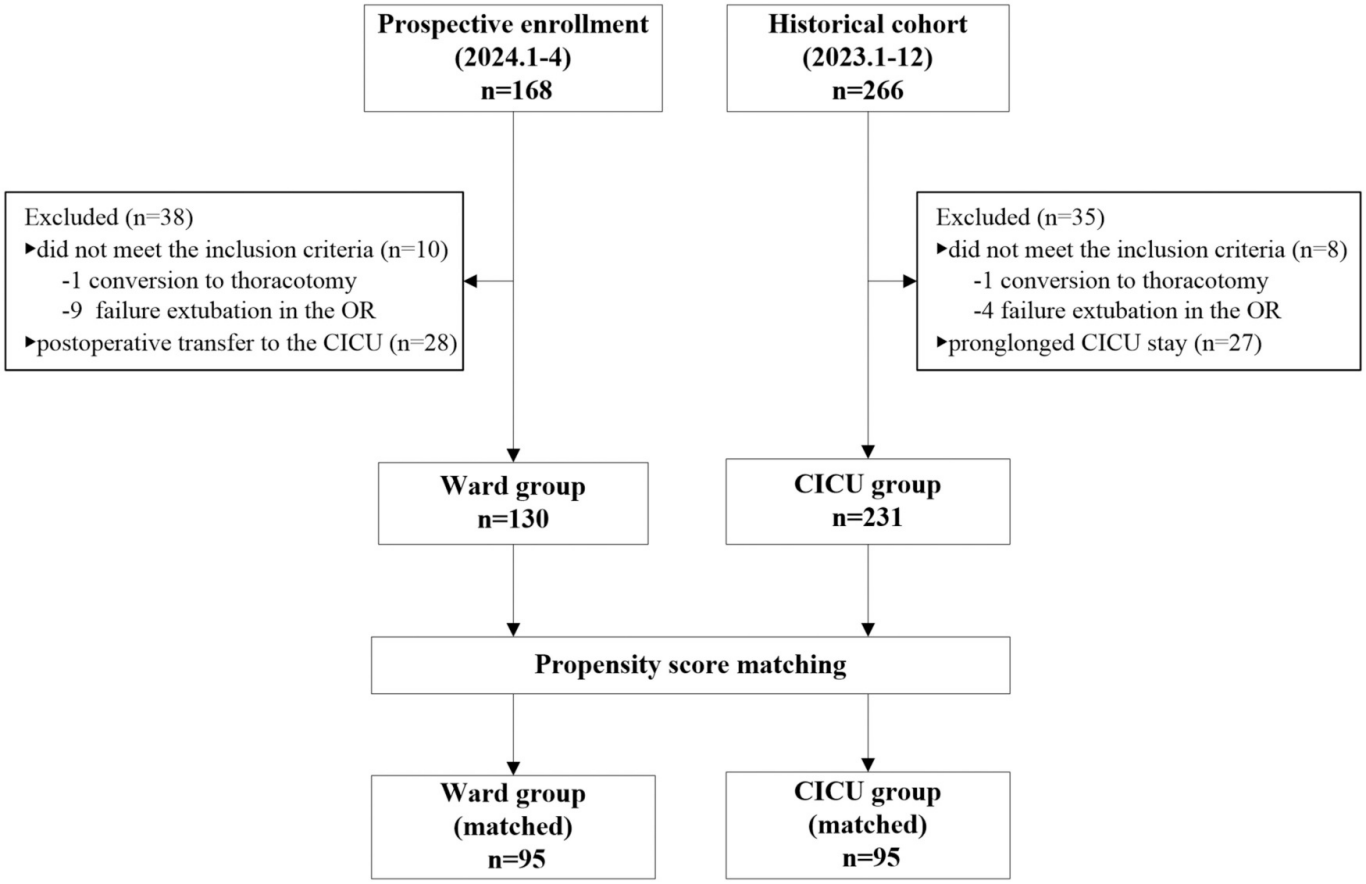
Flow diagram of the study. CICU, cardiac intensive care unit; OR, operating room.

A total of 168 patients underwent TAVR between January and April 2024. Patients were excluded for the following reasons: conversion to open thoracotomy (*n* = 1), failure or inability to achieve extubation in the operating room (*n* = 9), and postoperative transfer to the CICU (*n* = 28). Ultimately, 130 patients (77.4%) were directly transferred to the cardiovascular surgery ward following the procedure and were included in the analysis as the ward group (Figure 1).

In the historical cohort, 266 patients underwent TAVR between January and December 2023. Patients were excluded due to conversion to thoracotomy (*n* = 4), failure to extubate in the operating room (*n* = 4), or prolonged CICU stay exceeding 48 h (*n* = 27). The remaining 231 patients who were transferred to the CICU postoperatively were included as the CICU group (Figure 1).

Compared with patients in the CICU group, those directly admitted to the ward after TAVR were slightly younger (68 vs. 69 years, *P* = 0.020), had a lower prevalence of hypertension (48.5% vs. 60.2%, *P* = 0.031), a higher prevalence of malignancy (3.8% vs. 0.4%, *P* = 0.024), a greater proportion of transapical access (63.1% vs. 44.2%, *P* = 0.001), and a shorter procedure duration (44.5 vs. 50.0 min, *P* < 0.001) (Table 1). After propensity score matching, 95 patients were assigned to each group. Baseline characteristics of the matched cohorts were summarized in Table 1.

**Table 1:** Baseline demographic and clinical characteristics before and after propensity score matching.

Variables	Unmatached cohort			Matched cohort		
	Ward group (*n* = 130)	CICU group (*n* = 231)	*P*	Ward group (*n* = 95)	CICU group (*n* = 95)	*P*
Age, yrs*	68 (61, 72)	69 (65, 74)	0.020^a^	69 (61, 73)	68 (65, 72)	0.825^a^
Male (%)	86 (66.2)	136 (58.9)	0.172^c^	61 (64.2)	59 (62.1)	0.764^c^
BMI, kg/m^2^^#^	23.14 ± 3.01	22.91 ± 2.96	0.487^b^	22.9 ± 3.13	23.33 ± 3.10	0.360^b^
Current smoker (%)	9 (6.9)	15 (6.5)	0.875^c^	7 (7.4)	6 (6.3)	0.774^c^
NYHA functional class (%)			0.798^d^			0.271^d^
II	3 (2.3)	4 (1.7)		3 (3.2)	0	
III	61 (46.9)	116 (50.2)		46 (48.4)	45 (47.4)	
IV	66 (50.8)	111 (48.1)		46 (48.4)	50 (52.6)	
EuroSCORE II, %*	2.92 (1.92, 4.19)	3.42 (1.92, 5.26)	0.105^a^	2.93 (1.92, 4.63)	2.93 (1.93, 4.15)	0.893^a^
Left ventricular ejection fraction (%)			0.313^d^			0.303^d^
≥50%	110 (86.4)	194 (84.0)		82 (86.3)	85 (88.4)	
30%-50%	20 (15.4)	33 (14.3)		13 (13.7)	9 (9.5)	
<30%	0	4 (1.7)		0	2 (2.1)	
Comobidities (%)						
Hypertension	63 (48.5)	139 (60.2)	0.031^c^	50 (52.6)	47 (49.5)	0.663^c^
Diabetes	25 (19.2)	59 (25.5)	0.173^c^	17 (17.9)	14 (14.7)	0.556^c^
COPD	63 (48.2)	102 (44.3)	0.431^c^	43 (45.3)	44 (46.3)	0.884^c^
Chronic kidney disease	16 (12.3)	33 (14.3)	0.598^c^	11 (11.6)	9 (9.5)	0.636^c^
Cerebrovascular disease	29 (22.3)	55 (23.8)	0.746^c^	23 (24.2)	18 (18.9)	0.378^c^
Atrial fibrillation	13 (10.0)	21 (9.1)	0.777^c^	9 (9.5)	10 (10.5)	0.809^c^
Coronary artery disease	68 (52.3)	109 (47.2)	0.350^c^	47 (49.5)	42 (44.2)	0.467^c^
Peripheral vascular disease	68 (52.3)	106 (45.8)	0.241^c^	48 (50.5)	44 (46.3)	0.561^c^
Malignancy	5 (3.8)	1 (0.4)	0.024^d^	1 (1.1)	1 (1.1)	1.000^d^
Moderate + MV regurgitation	22 (16.9)	54 (23.4)	0.149^c^	17 (17.9)	17 (17.9)	1.000^c^
LBBB or RBBB (%)	8 (6.2)	13 (5.6)	0.838^c^	4 (4.2)	4 (4.2)	1.000^d^
Prior PCI (%)	3 (2.3)	6 (2.6)	1.000^d^	2 (2.1)	1 (1.1)	1.000^d^
Prior CABG (%)	1 (0.8)	1 (0.4)	1.000^d^	1 (1.1)	1 (1.1)	1.000^d^
Prior TAVR (%)	1 (0.8)	1 (0.4)	1.000^d^	1 (1.1)	0	1.000^d^
Prior SAVR (%)	1 (0.8)	3 (1.3)	1.000^d^	1 (1.1)	0	1.000^d^
Prior other caradic surgeries (%)	6 (4.6)	4 (1.7)	0.205^d^	2 (2.1)	2 (2.1)	1.000^d^
Prior PPM (%)	1 (0.8)	4 (1.7)	0.778^d^	1 (1.1)	1 (1.1)	1.000^d^
Aortic valve disease type (%)			0.868^c^			0.486^c^
AS	28 (21.5)	54 (23.4)		25 (26.3)	21 (22.1)	
AR	56 (43.1)	101 (43.7)		33 (34.7)	41 (43.2)	
Mixed AS/AR	46 (35.4)	76 (32.9)		37 (38.9)	33 (34.7)	
BAV (%)	9 (6.9)	16 (6.9)	0.999^c^	5 (5.3)	7 (7.4)	0.551^c^
Access (%)			0.001^c^			0.663^c^
Transfemoral	48 (36.9)	129 (55.8)		43 (45.3)	46 (48.4)	
Transapical	82 (63.1)	102 (44.2)		52 (54.7)	49 (51.6)	
Procedure time, min*	44.5 (36, 50)	50 (40, 60)	<0.001^a^	45 (37, 55)	50 (42, 57.5)	0.381^a^

IQR, interquartile range; CICU, cardiac intensive care unit; BMI, body mass index; NYHA, New York Heart Association; EuroSCORE II, European system for cardiac operative risk evaluation II; COPD, chronic obstructive pulmonary disease; MV, mitral valve; LBBB, left bundle branch block; RBBB, right bundle branch block; PCI, percutaneous coronary intervention; CABG, coronary artery bypass grafting; TAVR, transcatheter aortic valve replacement; SAVR, surgical aortic valve replacement; PPM, permanent pacemaker; AS, aortic stenosis; AR, aortic regurgitation; BAV, bicuspid aortic valve.

^a^ Statistical significance calculated with Mann–Whitney *U*-test.

^b^ Statistical significance calculated with independent two-sample *t*-test.

^c^ Statistical significance calculated with Pearson chi-squared test.

^d^ Statistical significance calculated with Fisher's exact test.

* Data are median (IQR),

# Data are mean ± standard deviation.

### Primary outcomes

A total of 39 patients (10.8%) experienced a VARC-3 early safety event within 30 days after TAVR. After propensity score matching, the incidence of early safety events was 10.0% (19 patients). In the matched cohort, 8.4% (8/95) of patients in the ward group experienced an event, compared with 11.6% (11/95) in the CICU group. Although the ward group showed a lower rate of composite safety events, the difference was not statistically significant (OR = 0.7, 95% CI: 0.3–1.7). Similar findings were observed in the unmatched cohort (Table 2).

**Table 2:** Comparison of study outcomes between the groups before and after propensity score matching.

Variables	Unmatached cohort				Matched cohort			
	Ward group (*n* = 130)	CICU group (*n* = 231)	Estimated Difference (95%CI)	*P*	Ward group (*n* = 95)	CICU group (*n* = 95)	Estimated Difference (95%CI)	*P*
Primary outcome								
Safety endpoints*	13 (10)	26 (11.3)	0.9 (0.5-1.7)	0.712^a^	8 (8.4)	11 (11.6)	0.7 (0.3-1.7)	0.468^a^
Secondary outcome								
All-cause death*	0	0	/	/	0	0	/	/
Cardiovascular death*	0	0	/	/	0	0	/	/
Stroke*	0	1 (0.4)	/	1.000^b^	0	1 (1.1)	/	1.000^b^
Bleeding type 2-4*	0	5 (2.2)	/	0.164	0	2 (2.1)	/	0.497^b^
Major vascular complications*	0	1 (0.4)	/	1.000^b^	0	1 (1.1)	/	1.000^b^
Major access related complications*	0	1 (0.4)	/	1.000^b^	0	0	/	/
Major cardiac structural complications*	0	0	/	/	0	0	/	/
Moderate or severe AR*	0	1 (0.4)	/	1.000^b^	0	0	/	/
New permanent pacemaker*	12 (9.2)	18 (7.8)	1.2 (0.6-2.6)	0.635^a^	8 (8.4)	6 (6.3)	1.4 (0.5-4.1)	0.579^a^
Acute kidney injury stage 3-4*	1 (0.8)	2 (0.9)	0.9 (0.1-9.9)	1.000^b^	0	2 (2.1)	/	0.497^b^
Myocardial infarction*	0	0	/	/	0	0	/	/
Postoperative delirium*	4 (3.1)	11 (4.8)	0.6 (0.2-2.0)	0.441^b^	2 (2.1)	5 (5.3)	0.4 (0.1-2.0)	0.444^b^
Secondary transfer to ICU*	0	0	/	/	0	0	/	/
Reintervention*	0	1 (0.4)	/	1.000^b^	0	0	/	/
Postoperative LOS, days^#^	4 (4, 5.3)	6 (5, 7)	1.6 (1.0-2.1)	<0.001^c^	4 (4, 5)	6 (5, 7)	1.9 (1.3-2.5)	<0.001^c^
Rehospitalization*	4 (3.1)	7 (3.0)	1.0 (0.3-3.5)	1.000^b^	2 (2.1)	2 (2.1)	1.0 (0.1-7.2)	1.000^b^

IQR, interquartile range; CI, confidence interval; CICU, cardiac intensive care unit; AR, aortic regurgitation; LOS, length of stay.

^a^ Statistical significance calculated with Pearson chi-squared test.

^b^ Statistical significance calculated with Fisher's exact test.

^c^ Statistical significance calculated with Mann–Whitney *U*-test.

* Data are n (%),

# Data are median (IQR).

### Second outcomes

Secondary outcomes at 30 days were presented in Table 2. Overall, 14 patients (7.4%) required a new permanent pacemaker implantation (PPI), including 8 patients (8.4%) in the ward group and 6 patients (6.3%) in the CICU group, with no statistically significant difference between groups (OR = 1.4, 95%CI: 0.5-4.1). Four patients were readmitted within 30 days: two for PPI, one for new-onset left bundle branch block (LBBB), and one for stroke. Notably, no cases of death, stroke, VARC type 2–4 bleeding, major vascular complications, access related complications, cardiac structural complications, moderate or severe AR, acute kidney injury stage 3-4, or myocardial infarction were reported in the ward group, whereas several such events occurred in the CICU group. However, none of these differences reached statistical significance. Furthermore, no patients in either group required device-related reintervention or secondary transfer to ICU. Although postoperative delirium occurred more frequently in the CICU group, the difference was not statistically significant. Finally, the median postoperative LOS was significantly shorter in the ward group (4 days, IQR: 4-5) compared with the CICU group (6 days, IQR: 5-7), with a mean difference of 1.9 days (95%CI: 1.3-2.5). Similar results were observed in the unmatched cohort (Table 2). Additional comparisons stratified by valve disease type are presented in Supplementary Tables S1–S6.

## Discussions

The primary findings of this study are summarized as follows: (1) Nearly 80% of patients were directly admitted to the cardiovascular surgery ward following TAVR; (2) direct transfer to the ward in selected patients appeared safe and feasible, with no increased risks of postoperative complications, unplanned ICU transfers, 30-day readmissions, or mortality compared with conventional CICU admission; (3) direct ward admission was associated with a shorter postoperative length of stay.

Previous studies have demonstrated the safety of direct ward transfer following TAVR, albeit predominantly in carefully selected patient populations. Leclercq et al. (17) were among the first to propose a comprehensive 14-item risk stratification protocol to identify suitable candidates. The protocol incorporated factors such as left ventricular ejection fraction (LVEF), baseline conduction abnormalities, transfemoral access, hemodynamic state, and the absence of per and immediate postprocedural events. Patients classified as low-risk were transferred directly to the ward, while high-risk patients were admitted to the ICU. Interestingly, 25.8% were categorized as low-risk, yet 14.1% of high-risk patients were nonetheless transferred directly to the ward (18), suggesting that the protocol, while thorough, may be somewhat conservative in routine clinical practice. Cohen et al. (19) later introduced a simplified set of four criteria focusing on periprocedural hemodynamics and complications, though the retrospective nature of their study may limit its generalizability. In our study, we incorporated respiratory support status in addition to perioperative hemodynamic stability and the absence of complications. Preoperative respiratory support may indicate impaired cardiopulmonary function, while immediate postoperative extubation is particularly relevant in our center, where transapical TAVR—commonly used for aortic regurgitation—requires general anesthesia and endotracheal intubation. Although conscious sedation or monitored anesthesia care is increasingly adopted in transfemoral TAVR (21, 22), transapical access remains more complex. Our criteria may thus provide practical guidance for evaluating such cases. Notably, nearly 80% of patients in our cohort were safely transferred directly to the general ward—a proportion higher than previously reported (17, 18). This may be partly attributed to the broader risk spectrum of our population, which included lower-risk patients with lower EuroSCOREs than those in Leclercq et al.'s study. These findings remain clinically relevant, particularly as TAVR is increasingly performed in intermediate- and low-risk populations.

The overall incidence of VARC-3 early safety events was low, and most specific complications were infrequent. Permanent pacemaker implantation (PPI) was the most common complication, occurring in 8.3% of patients—a rate generally consistent with previous TAVR studies (23–25). No statistically significant differences in early safety events or specific complications were observed between patients directly returned to the ward and those admitted to the CICU, in both matched and unmatched cohorts. To ensure patient safety during early postoperative ward recovery after TAVR, we implemented a three-enhanced ward monitoring protocol comprising enhanced observations, enhanced examinations, and enhanced manpower. These measures reflect our center's emphasis on close monitoring and may have contributed to the favorable outcome observed. Notably, the CICU group was retrospectively identified, and a two-step process was used to ensure comparability: first, applying the same inclusion criteria as the ward group, with an additional restriction of ICU length of stay ≤48 h to minimize selection bias and exclude patients who were either too sick for direct ward transfer or who deteriorated during their ICU stay; and second, performing propensity score matching to balance baseline characteristics. These steps help improve comparability and interpretability.

As expected, our findings showed that patients directly returned to the general ward after TAVR had a shorter postoperative length of stay compared to those admitted to the CICU (median 4 vs. 6 days, *P* < 0.001). This may be partly explained by the fact that bypassing ICU admission avoids the additional time typically spent in intensive care and the subsequent transition back to the ward, which often resets postoperative routines such as vital sign monitoring and medication adjustments. Moreover, recovering in the ward —characterized by reduced nighttime disruptions, less invasive monitoring, and greater access to family support—may encourage earlier ambulation and rehabilitation, both of which are the key to functional recovery after TAVR (26, 27). Although the incidence of postoperative delirium was lower in the ward group (2.1% vs. 5.3%), the difference did not reach statistical significance (*P* = 0.444), possibly due to the low event rate or limited sample size. Nonetheless, this trend is worth further exploration. Notably, no patients in either group required ICU readmission, further supporting the safety of direct ward admission in appropriately selected patients.

## Limitations

This study has several limitations. First, although patients in the ward group were prospectively enrolled, the CICU group was retrospectively selected. Despite efforts to apply the same inclusion criteria, restrict ICU stay to ≤48 h, and perform propensity score matching to reduce confounding, the potential for selection bias and residual confounders remains. Second, the relatively small sample size—particularly for infrequent events such as postoperative complications—may have limited the statistical power to detect between-group differences. Third, this was a single-center study with a unique clinical profile. Notably, a relatively high proportion of procedures were performed via the transapical approach, mainly reflecting the high representation of pure AR and mixed AS/AR in our cohort and the preferential use of a transapical strategy in selected AR-related cases during the study period (e.g., J-Valve). Moreover, because a higher transapical proportion may be associated with more complex perioperative care needs, the observed feasibility in our cohort may cautiously and indirectly support its applicability in predominantly transfemoral practice. Along with the broader baseline risk spectrum, these factors may limit the generalizability of our findings. Finally, long-term outcomes such as functional recovery and quality of life were not assessed, and further prospective studies with extended follow-up are warranted.

## Conclusions

Direct transfer to the cardiovascular surgery ward after TAVR appears safe and feasible for appropriately selected patients and is associated with a shorter postoperative length of stay compared to routine CICU admission. These findings support the use of risk-adapted postoperative care pathways to optimize recovery and resource utilization.

## Data Availability

The raw data supporting the conclusions of this article will be made available by the authors, without undue reservation.
