# Supplementary material for: Safety and feasibility of direct return to the ward after transcatheter aortic valve replacement: a prospective observational study
Source: Front Cardiovasc Med. 2026 Mar 11;13:1773793. doi: 10.3389/fcvm.2026.1773793 (PMC13013441; doi:10.3389/fcvm.2026.1773793)
Supplement: Supplementary file 1 [file Datasheet1.docx]

**Supplementary Table 1** Baseline Demographic and Clinical Characteristics in the AS Subgroup Before and after Propensity Score Matching

| **Variables** | **Unmatached Cohort** | | | **Matched Cohort** | | |
| --- | --- | --- | --- | --- | --- | --- |
|  | **Ward group (n=28)** | **CICU group (n=54)** | ***P*** | **Ward group (n=25)** | **CICU group (n=21)** | ***P*** |
| Age, yrs* | 69 (60.3, 72) | 71.5 (64.5, 74) | 0.208^a^ | 69 (60, 72) | 69 (62.5, 73) | 0.724^a^ |
| Male (%) | 15 (53.6) | 30 (55.6) | 1.000^c^ | 12 (48.0) | 14 (66.7) | 0.244^c^ |
| BMI, kg/m^2#^ | 22.41±2.86 | 23.10±3.10 | 0.331^b^ | 22.57±2.96 | 23.99±3.22 | 0.127^b^ |
| Current smoker (%) | 1 (3.6) | 5 (9.3) | 0.427^d^ | 1 (4.0) | 2 (9.5) | 0.585^d^ |
| NYHA functional class (%) |  |  | 0.668^d^ |  |  | 0.072^d^ |
| Ⅱ | 0 | 2 (3.7) |  | 0 | 0 |  |
| Ⅲ | 18 (64.3) | 27 (50.0) |  | 18 (72.0) | 9 (42.9) |  |
| Ⅳ | 10 (35.7) | 25 (46.3) |  | 7 (28.0) | 12 (57.1) |  |
| EuroSCORE II, %* | 3.00 (1.46, 4.13) | 3.65 (2.14, 6.76) | 0.172^a^ | 2.60 (2.73) | 2.75 (4.86) | 0.903^a^ |
| Left ventricular ejection fraction (%) | |  | 0.313^d^ |  |  | 0.723^d^ |
| ≥50% | 25 (89.3) | 48 (88.9) |  | 24 (96.0) | 19 (90.5) |  |
| 30%-50% | 3 (10.0) | 4 (7.4) |  | 1 (4.0) | 1 (4.8) |  |
| <30% | 0 | 2 (3.7) |  | 0 | 1 (4.8) |  |
| Comobidities (%) |  |  |  |  |  |  |
| Hypertension | 10 (35.7) | 24 (44.4) | 0.487^c^ | 10 (40.0) | 7 (33.3) | 0.762^c^ |
| Diabetes | 7 (25.0) | 19 (35.2) | 0.455^c^ | 6 (24.0) | 3 (14.3) | 0.478^d^ |
| COPD | 13 (46.4) | 26 (48.1) | 0.979^c^ | 11 (44.0) | 9 (42.9) | 0.928^c^ |
| Chronic kidney disease | 1 (3.6) | 6 (11.1) | 0.413^d^ | 1 (4.0) | 0 | 1.000^d^ |
| Cerebrovascular disease | 2 (7.1) | 9 (16.7) | 0.316^d^ | 2 (8.0) | 1 (4.8) | 1.000^d^ |
| Atrial fibrillation | 2 (7.1) | 5 (9.3) | 1.000^d^ | 2 (8.0) | 3 (14.3) | 0.648^d^ |
| Coronary artery disease | 12 (42.9) | 27 (50.0) | 0.643^c^ | 9 (36.0) | 7 (33.3) | 1.000^c^ |
| Peripheral vascular disease | 14 (50.0) | 26 (48.1) | 0.770^c^ | 13 (52.0) | 9 (42.9) | 0.536^c^ |
| Malignancy | 1 (3.6) | 0 | 0.341^d^ | 0 | 0 | / |
| Moderate+ MV regurgitation | 3 (10.7) | 11 (20.4) | 0.361^d^ | 3 (12.0) | 3 (14.3) | 1.000^d^ |
| LBBB or RBBB (%) | 1 (3.6) | 2 (3.7) | 1.000^d^ | 0 | 0 | / |
| Prior PCI (%) | 0 | 1 (1.9) | 1.000^d^ | 0 | 1 (4.8) | 0.457^d^ |
| Prior CABG (%) | 1 (3.6) | 1 (1.9) | 1.000^d^ | 1 (4.0) | 1 (4.8) | 1.000^d^ |
| Prior TAVR (%) | 0 | 0 | / | 0 | 0 | / |
| Prior SAVR (%) | 1 (1.9) | 1 (3.6) | 1.000^d^ | 1 (4.0) | 0 | 1.000^d^ |
| Prior other caradic surgeries (%) | 0 | 1 (1.9) | 1.000^d^ | 0 | 0 | / |
| Prior PPM (%) | 0 | 1 (1.9) | 1.000^d^ | 0 | 0 | / |
| BAV (%) | 4 (14.3) | 9 (16.7) | 1.000^d^ | 3 (12.0) | 2 (9.5) | 1.000^d^ |
| Access (%) |  |  | 1.000^d^ |  |  | 1.000^d^ |
| Transfemoral | 28 (100) | 54 (100) |  | 25 (100) | 21 (100) |  |
| Transapical | 0 | 0 |  | 0 | 0 |  |
| Procedure time, min* | 45 (30.8, 58.8) | 50 (40.8, 60) | 0.210^a^ | 45 (30, 62.5) | 51 (43, 60) | 0.234^a^ |

*Data are median (IQR), ^#^Data are mean ± standard deviation

Abbreviations: IQR, interquartile range; CICU, cardiac intensive care unit; BMI, body mass index; NYHA, New York Heart Association; EuroSCORE II, European system for cardiac operative risk evaluation II; COPD, chronic obstructive pulmonary disease; MV, mitral valve; LBBB, left bundle branch block; RBBB, right bundle branch block; PCI, percutaneous coronary intervention; CABG, coronary artery bypass grafting; TAVR, transcatheter aortic valve replacement; SAVR, surgical aortic valve replacement; PPM, permanent pacemaker; AS, aortic stenosis; AR, aortic regurgitation; BAV, bicuspid aortic valve.

^a^ Statistical significance calculated with Mann-Whitney *U*-test.

^b^ Statistical significance calculated with independent two-sample *t*-test.

^c^ Statistical significance calculated with Pearson chi-squared test.

^d^ Statistical significance calculated with Fisher’s exact test.

**Supplementary Table 2** Baseline Demographic and Clinical Characteristics in the AR Subgroup Before and after Propensity Score Matching

| **Variables** | **Unmatached Cohort** | | | **Matched Cohort** | | |
| --- | --- | --- | --- | --- | --- | --- |
|  | **Ward group (n=56)** | **CICU group (n=101)** | ***P*** | **Ward group (n=33)** | **CICU group (n=41)** | ***P*** |
| Age, yrs* | 68 (65, 72.8) | 69 (66, 75) | 0.241^a^ | 70 (66.5, 74.5) | 68 (65.5, 71.5) | 0.230^a^ |
| Male (%) | 42 (75.0) | 67 (66.3) | 0.283^c^ | 25 (75.8) | 29 (70.7) | 0.793^c^ |
| BMI, kg/m^2#^ | 23.17±3.21 | 22.76±3.04 | 0.435^b^ | 22.63±3.22 | 23.03±3.27 | 0.600^b^ |
| Current smoker (%) | 5 (8.9) | 6 (5.9) | 0.523^c^ | 3 (9.1) | 3 (7.3) | 1.000^d^ |
| NYHA functional class (%) |  |  | 0.880^d^ |  |  | 0.242^d^ |
| Ⅱ | 2 (3.6) | 2 (2.0) |  | 2 (6.1) | 0 |  |
| Ⅲ | 23 (41.1) | 47 (46.5) |  | 12 (36.4) | 19 (46.3) |  |
| Ⅳ | 31 (55.4) | 52 (51.5) |  | 19 (57.6) | 22 (53.7) |  |
| EuroSCORE II, %* | 2.97 (1.92, 4.73) | 3.42 (1.54, 5.12) | 0.593^a^ | 3.07 (1.95, 5.05) | 3.09 (1.91, 3.90) | 0.463^a^ |
| Left ventricular ejection fraction (%) | |  | 0.484^d^ |  |  | 0.119^d^ |
| ≥50% | 43 (76.8) | 83 (82.2) |  | 25 (75.8) | 36 (87.8) |  |
| 30%-50% | 13 (23.2) | 17 (16.8) |  | 8 (24.2) | 4 (9.8) |  |
| <30% | 0 | 1 (1.0) |  | 0 | 1 (2.4) |  |
| Comobidities (%) |  |  |  |  |  |  |
| Hypertension | 30 (53.6) | 79 (78.2) | 0.001^c^ | 19 (57.6) | 29 (70.7) | 0.328^c^ |
| Diabetes | 22 (21.8) | 22 (21.8) | 0.680^c^ | 5 (15.2) | 8 (19.5) | 0.762^c^ |
| COPD | 28 (50.0) | 44 (43.6) | 0.439^c^ | 16 (48.5) | 18 (43.9) | 0.694^c^ |
| Chronic kidney disease | 9 (16.1) | 16 (15.8) | 1.000^c^ | 4 (12.1) | 6 (14.6) | 1.000^d^ |
| Cerebrovascular disease | 19 (33.9) | 25 (24.8) | 0.266^c^ | 13 (39.4) | 9 (22.0) | 0.128^c^ |
| Atrial fibrillation | 8 (14.3) | 10 (9.9) | 0.440^c^ | 6 (18.2) | 3 (7.3) | 0.283^d^ |
| Coronary artery disease | 34 (60.7) | 49 (48.5) | 0.182^c^ | 21 (63.6) | 19 (46.3) | 0.164^c^ |
| Peripheral vascular disease | 29 (51.9) | 45 (44.6) | 0.230^c^ | 16 (48.5) | 19 (46.3) | 0.856^c^ |
| Malignancy | 4 (7.1) | 0 | 0.015^d^ | 1 (3.0) | 0 | 0.446^d^ |
| Moderate+ MV regurgitation | 10 (17.9) | 26 (25.7) | 0.323^c^ | 7 (21.2) | 9 (22.0) | 1.000^c^ |
| LBBB or RBBB (%) | 4 (7.1) | 8 (7.9) | 1.000^d^ | 2 (6.1) | 2 (4.9) | 1.000^d^ |
| Prior PCI (%) | 2 (3.6) | 4 (4.0) | 1.000^d^ | 2 (6.1) | 0 | 0.195^d^ |
| Prior CABG (%) | 0 | 0 | / | 0 | 0 | / |
| Prior TAVR (%) | 1 (1.3) | 1 (1.0) | 1.000^d^ | 1 (3.0) | 0 | 0.443^d^ |
| Prior SAVR (%) | 0 | 2 (2.0) | 1.000^d^ | 0 | 0 | / |
| Prior other caradic surgeries (%) | 2 (3.6) | 1 (1.0) | 1.000^d^ | 0 | 0 | / |
| Prior PPM (%) | 0 | 2 (2.0) | 1.000^d^ | 0 | 0 | / |
| BAV (%) | 2 (3.6) | 4 (4.0) | 1.000^d^ | 1 (3.0) | 3 (7.3) | 0.624^d^ |
| Access (%) |  |  | 0.386^c^ |  |  | 0.634^c^ |
| Transfemoral | 8 (14.3) | 20 (19.8) |  | 5 (15.2) | 8 (19.5) |  |
| Transapical | 48 (85.7) | 81 (80.2) |  | 28 (84.8) | 33 (80.5) |  |
| Procedure time, min* | 46.5 (41.3, 50) | 46 (40, 57) | 0.409^a^ | 48 (42, 53) | 44 (40, 53.5) | 0.181^a^ |

*Data are median (IQR), ^#^Data are mean ± standard deviation

Abbreviations: IQR, interquartile range; CICU, cardiac intensive care unit; BMI, body mass index; NYHA, New York Heart Association; EuroSCORE II, European system for cardiac operative risk evaluation II; COPD, chronic obstructive pulmonary disease; MV, mitral valve; LBBB, left bundle branch block; RBBB, right bundle branch block; PCI, percutaneous coronary intervention; CABG, coronary artery bypass grafting; TAVR, transcatheter aortic valve replacement; SAVR, surgical aortic valve replacement; PPM, permanent pacemaker; AS, aortic stenosis; AR, aortic regurgitation; BAV, bicuspid aortic valve.

^a^ Statistical significance calculated with Mann-Whitney *U*-test.

^b^ Statistical significance calculated with independent two-sample *t*-test.

^c^ Statistical significance calculated with Pearson chi-squared test.

^d^ Statistical significance calculated with Fisher’s exact test.

**Supplementary Table 3** Baseline Demographic and Clinical Characteristics in the Mixed AS/AR Subgroup Before and after Propensity Score Matching

| **Variables** | **Unmatached Cohort** | | | **Matched Cohort** | | |
| --- | --- | --- | --- | --- | --- | --- |
|  | **Ward group (n=46)** | **CICU group (n=76)** | ***P*** | **Ward group (n=37)** | **CICU group (n=33)** | ***P*** |
| Age, yrs* | 66.5 (60, 73) | 69 (65, 73) | 0.131^a^ | 67 (60, 73) | 68 (65, 72.5) | 0.604^a^ |
| Male (%) | 29 (63.0) | 39 (51.3) | 0.260^c^ | 24 (64.9) | 16 (48.5) | 0.227^c^ |
| BMI, kg/m^2#^ | 23.54±3.05 | 22.96±2.78 | 0.285^b^ | 23.39±3.18 | 23.26±2.82 | 0.869^b^ |
| Current smoker (%) | 3 (6.5) | 4 (5.3) | 1.000^d^ | 3 (8.1) | 1 (3.0) | 0.616^d^ |
| NYHA functional class (%) |  |  | 0.442^d^ |  |  | 0.717^d^ |
| Ⅱ | 1 (2.2) | 0 |  | 1 (2.7) | 0 |  |
| Ⅲ | 20 (43.5) | 42 (55.3) |  | 16 (43.2) | 17 (51.5) |  |
| Ⅳ | 25 (54.3) | 34 (44.7) |  | 20 (54.1) | 16 (48.5) |  |
| EuroSCORE II, %* | 2.73 (1.98, 4.05) | 3.33 (2.04, 4.98) | 0.281^a^ | 2.73 (1.95, 4.22) | 2.99 (2.32, 4.70) | 0.459^a^ |
| Left ventricular ejection fraction (%) | |  | 0.379^d^ |  |  | 1.000^d^ |
| ≥50% | 42 (91.3) | 63 (82.9) |  | 33 (89.1) | 29 (87.9) |  |
| 30%-50% | 4 (8.7) | 12 (15.8) |  | 4 (10.8) | 4 (12.1) |  |
| <30% | 0 | 1 (1.3) |  | 0 | 0 |  |
| Comobidities (%) |  |  |  |  |  |  |
| Hypertension | 23 (50.0) | 36 (47.4) | 0.852^c^ | 21 (56.8) | 11 (33.3) | 0.059^c^ |
| Diabetes | 8 (17.4) | 18 (23.7) | 0.497^c^ | 6 (16.2) | 3 (9.1) | 0.485^d^ |
| COPD | 22 (47.8) | 32 (42.1) | 0.538^c^ | 16 (43.2) | 17 (51.5) | 0.489^c^ |
| Chronic kidney disease | 6 (13.0) | 11 (14.5) | 1.000^c^ | 11 (11.6) | 9 (9.5) | 0.636^c^ |
| Cerebrovascular disease | 8 (17.4) | 21 (27.6) | 0.273^c^ | 8 (21.6) | 8 (24.2) | 1.000^c^ |
| Atrial fibrillation | 3 (6.5) | 6 (7.9) | 1.000^d^ | 1 (2.7) | 4 (12.1) | 0.181^d^ |
| Coronary artery disease | 22 (47.8) | 33 (43.4) | 0.709^c^ | 17 (45.9) | 16 (48.5) | 1.000^c^ |
| Peripheral vascular disease | 25 (54.3) | 35 (46.1) | 0.374^c^ | 19 (51.4) | 16 (48.5) | 0.811^c^ |
| Malignancy | 0 | 1 (1.3) | 1.000^d^ | 0 | 1 (3.0) | 0.471^d^ |
| Moderate+ MV regurgitation | 9 (19.6) | 17 (22.4) | 0.821^c^ | 7 (18.9) | 5 (15.2) | 0.758^c^ |
| LBBB or RBBB (%) | 3 (6.5) | 2 (3.7) | 1.000^d^ | 2 (5.4) | 2 (6.1) | 1.000^d^ |
| Prior PCI (%) | 1 (2.2) | 1 (1.3) | 1.000^d^ | 0 | 0 | / |
| Prior CABG (%) | 0 | 0 | / | 0 | 0 | / |
| Prior TAVR (%) | 0 | 0 | / | 0 | 0 | / |
| Prior SAVR (%) | 0 | 0 | / | 0 | 0 | / |
| Prior other caradic surgeries (%) | 4 (8.7) | 2 (2.6) | 0.197^d^ | 2 (5.4) | 2 (6.1) | 1.000^d^ |
| Prior PPM (%) | 1 (2.2) | 1 (1.3) | 1.000^d^ | 1 (2.7) | 1 (3.0) | 1.000^d^ |
| BAV (%) | 3 (6.5) | 3 (3.9) | 0.671^d^ | 1 (2.7) | 2 (6.1) | 0.599^d^ |
| Access (%) |  |  | <0.001^c^ |  |  | 0.167^c^ |
| Transfemoral | 12 (26.1) | 55 (72.4) |  | 13 (35.1) | 17 (51.5) |  |
| Transapical | 34 (73.9) | 21 (27.6) |  | 24 (64.9) | 16 (48.5) |  |
| Procedure time, min* | 40 (34.5, 49.2) | 53.5 (44.3, 64) | <0.001^a^ | 40 (35, 50.5) | 53 (44.5, 64.5) | 0.004^a^ |

*Data are median (IQR), ^#^Data are mean ± standard deviation

Abbreviations: IQR, interquartile range; CICU, cardiac intensive care unit; BMI, body mass index; NYHA, New York Heart Association; EuroSCORE II, European system for cardiac operative risk evaluation II; COPD, chronic obstructive pulmonary disease; MV, mitral valve; LBBB, left bundle branch block; RBBB, right bundle branch block; PCI, percutaneous coronary intervention; CABG, coronary artery bypass grafting; TAVR, transcatheter aortic valve replacement; SAVR, surgical aortic valve replacement; PPM, permanent pacemaker; AS, aortic stenosis; AR, aortic regurgitation; BAV, bicuspid aortic valve.

^a^ Statistical significance calculated with Mann-Whitney *U*-test.

^b^ Statistical significance calculated with independent two-sample *t*-test.

^c^ Statistical significance calculated with Pearson chi-squared test.

^d^ Statistical significance calculated with Fisher’s exact test.

**Supplementary Table 4** Comparison of Study Outcomes Between the Groups in the AS Subgroup Before and After Propensity Score Matching

| **Variables** | **Unmatached Cohort** | | | | **Matched Cohort** | | | |
| --- | --- | --- | --- | --- | --- | --- | --- | --- |
|  | **Ward group (n=28)** | **CICU group (n=54)** | **Estimated Difference (95%CI)** | ***P*** | **Ward group (n=25)** | **CICU group (n=21)** | **Estimated Difference (95%CI)** | ***P*** |
| Primary outcome |  |  |  |  |  |  |  |  |
| Safety endpoints* | 4 (14.3) | 2 (3.7) | 0.2 (0.04-1.3) | 0.174^a^ | 4 (16.0) | 0 | / | 0.114^a^ |
| Secondary outcome |  |  |  |  |  |  |  |  |
| All-cause death* | 0 | 0 | / | / | 0 | 0 | / | / |
| Cardiovascular death* | 0 | 0 | / | / | 0 | 0 | / | / |
| Stroke* | 0 | 0 | / | / | 0 | 0 | / | / |
| Bleeding type 2-4* | 0 | 0 | / | / | 0 | 0 | / | / |
| Major vascular complications* | 0 | 0 | / | / | 0 | 0 | / | / |
| Major access related complications* | 0 | 0 | / | / | 0 | 0 | / | / |
| Major cardiac structural complications* | 0 | 0 | / | / | 0 | 0 | / | / |
| Moderate or severe AR* | 0 | 1 (1.9) | / | 1.000^a^ | 0 | 0 | / | / |
| New permanent pacemaker* | 4 (14.3) | 1 (1.9) | 8.8 (0.9-83.3) | 0.044^a^ | 4 (16.0) | 0 | / | 0.114^a^ |
| Acute kidney injury stage 3-4* | 0 | 0 | / | / | 0 | 0 | / | / |
| Myocardial infarction* | 0 | 0 | / | / | 0 | 0 | / | / |
| Postoperative delirium* | 1 (3.6) | 1 (1.9) | 2.0 (0.1-32.6) | 1.000^a^ | 0 | 1 (4.8) | / | 0.457^a^ |
| Secondary transfer to ICU* | 0 | 0 | / | / | 0 | 0 | / | / |
| Reintervention* | 0 | 1 (1.9) | / | 1.000^a^ | 0 | 0 | / | / |
| Postoperative LOS, days^#^ | 3.5 (3, 6) | 5 (4, 6.25) | 1.5 (0.3-2.7) | 0.001^b^ | 3 (3, 5.5) | 6 (5, 7) | 1.9 (0.6-3.2) | 0.001^b^ |
| Rehospitalization* | 1 (3.6) | 0 | / | 0.342^a^ | 1 (4.0) | 0 | / | 1.000^a^ |

*Data are n (%), ^#^Data are median (IQR)

Abbreviations: IQR, interquartile range; CI, confidence interval; CICU, cardiac intensive care unit; AR, aortic regurgitation; LOS, length of stay

^a^ Statistical significance calculated with Fisher’s exact test.

^b^ Statistical significance calculated with Mann-Whitney *U*-test.

**Supplementary Table 5** Comparison of Study Outcomes Between the Groups in the AR Subgroup Before and After Propensity Score Matching

| **Variables** | **Unmatached Cohort** | | | | **Matched Cohort** | | | |
| --- | --- | --- | --- | --- | --- | --- | --- | --- |
|  | **Ward group (n=56)** | **CICU group (n=101)** | **Estimated Difference (95%CI)** | ***P*** | **Ward group (n=33)** | **CICU group (n=41)** | **Estimated Difference (95%CI)** | ***P*** |
| Primary outcome |  |  |  |  |  |  |  |  |
| Safety endpoints* | 4 (7.1) | 17 (16.8) | 2.6 (0.8-8.2) | 0.140^a^ | 1 (3.0) | 6 (14.6) | 5.5 (0.6-48.1) | 0.123^a^ |
| Secondary outcome |  |  |  |  |  |  |  |  |
| All-cause death* | 0 | 0 | / | / | 0 | 0 | / | / |
| Cardiovascular death* | 0 | 0 | / | / | 0 | 0 | / | / |
| Stroke* | 0 | 0 | / | / | 0 | 0 | / | / |
| Bleeding type 2-4* | 0 | 4 (4.0) | / | 0.298^a^ | 0 | 1 (2.4) | / | 1.000^a^ |
| Major vascular complications* | 0 | 0 | / | / | 0 | 0 | / | / |
| Major access related complications* | 0 | 1 (1.0) | / | 1.000^a^ | 0 | 0 | / | / |
| Major cardiac structural complications* | 0 | 0 | / | / | 0 | 0 | / | / |
| Moderate or severe AR* | 0 | 0 | / | / | 0 | 0 | / | / |
| New permanent pacemaker* | 3 (5.4) | 13 (12.9) | 0.4 (0.1-1.4) | 0.174^a^ | 1 (3.0) | 4 (9.8) | 0.3 (0.03-2.7) | 0.373^a^ |
| Acute kidney injury stage 3-4* | 1 (1.8) | 1 (1.0) | 1.8 (0.1-29.6) | 1.000^a^ | 0 | 1 (2.4) | / | 1.000^a^ |
| Myocardial infarction* | 0 | 0 | / | / | 0 | 0 | / | / |
| Postoperative delirium* | 2 (3.6) | 7 (6.9) | 0.5 (0.1-2.5) | 0.492^a^ | 1 (3.0) | 2 (4.9) | 0.6 (0.05-7.0) | 1.000^a^ |
| Secondary transfer to ICU* | 0 | 0 | / | / | 0 | 0 | / | / |
| Reintervention* | 0 | 0 | / | / | 0 | 0 | / | / |
| Postoperative LOS, days^#^ | 5 (4, 5.75) | 6 (5, 8) | 1.8 (0.9-2.6) | <0.001^b^ | 5 (4, 6) | 6 (5, 8) | 1.9 (0.9-3.0) | <0.001^b^ |
| Rehospitalization* | 1 (1.8) | 4 (4.0) | 0.4 (0.05-4.0) | 0.656^a^ | 0 | 0 | / | / |

*Data are n (%), ^#^Data are median (IQR)

Abbreviations: IQR, interquartile range; CI, confidence interval; CICU, cardiac intensive care unit; AR, aortic regurgitation; LOS, length of stay

^a^ Statistical significance calculated with Fisher’s exact test.

^b^ Statistical significance calculated with Mann-Whitney *U*-test.

**Supplementary Table 6** Comparison of Study Outcomes Between the Groups in the Mixed AS/AR Subgroup Before and After Propensity Score Matching

| **Variables** | **Unmatached Cohort** | | | | **Matched Cohort** | | | |
| --- | --- | --- | --- | --- | --- | --- | --- | --- |
|  | **Ward group (n=46)** | **CICU group (n=76)** | **Estimated Difference (95%CI)** | ***P*** | **Ward group (n=37)** | **CICU group (n=33)** | **Estimated Difference (95%CI)** | ***P*** |
| Primary outcome |  |  |  |  |  |  |  |  |
| Safety endpoints* | 5 (10.9) | 7 (9.2) | 0.8 (0.2-2.8) | 1.000^a^ | 3 (8.1) | 5 (15.2) | 2.0 (0.4-9.2) | 0.462^a^ |
| Secondary outcome |  |  |  |  |  |  |  |  |
| All-cause death* | 0 | 0 | / | / | 0 | 0 | / | / |
| Cardiovascular death* | 0 | 0 | / | / | 0 | 0 | / | / |
| Stroke* | 0 | 1 (1.3) | / | 1.000^a^ | 0 | 1 (3.0) | / | 0.471^a^ |
| Bleeding type 2-4* | 0 | 1 (1.3) | / | 1.000^a^ | 0 | 1 (3.0) | / | 0.471^a^ |
| Major vascular complications* | 0 | 1 (1.3) | / | 1.000^a^ | 0 | 1 (3.0) | / | 0.471^a^ |
| Major access related complications* | 0 | 0 | / | / | 0 | 0 | / | / |
| Major cardiac structural complications* | 0 | 0 | / | / | 0 | 0 | / | / |
| Moderate or severe AR* | 0 | 0 | / | / | 0 | 0 | / | / |
| New permanent pacemaker* | 5 (10.9) | 4 (5.3) | 2.2 (0.5-8.6) | 0.295^a^ | 3 (8.1) | 2 (6.1) | 1.4 (0.2-8.7) | 1.000^a^ |
| Acute kidney injury stage 3-4* | 0 | 1 (1.3) | / | 1.000^a^ | 0 | 1 (3.0) | / | 0.471^a^ |
| Myocardial infarction* | 0 | 0 | / | / | 0 | 0 | / | / |
| Postoperative delirium* | 1 (2.2) | 3 (3.9) | 0.5 (0.1-5.4) | 1.000^a^ | 1 (2.7) | 2 (6.1) | 0.4 (0.04-5.0) | 0.599^a^ |
| Secondary transfer to ICU* | 0 | 0 | / | / | 0 | 0 | / | / |
| Reintervention* | 0 | 0 | / | / | 0 | 0 | / | / |
| Postoperative LOS, days^#^ | 4 (4, 5) | 6 (5, 7) | 1.4 (0.5-2.2) | <0.001^b^ | 4 (3.5, 5) | 6 (5, 7) | 1.6 (0.8-2.5) | <0.001^b^ |
| Rehospitalization* | 2 (4.1) | 3 (3.9) | 1.1 (0.2-6.9) | 1.000^a^ | 1 (2.7) | 2 (6.1) | 0.4 (0.04-5.0) | 0.599^a^ |

*Data are n (%), ^#^Data are median (IQR)

Abbreviations: IQR, interquartile range; CI, confidence interval; CICU, cardiac intensive care unit; AR, aortic regurgitation; LOS, length of stay

^a^ Statistical significance calculated with Fisher’s exact test.

^b^ Statistical significance calculated with Mann-Whitney *U*-test.
